# Supplementary material for: Gene Expression Profiles of Methyltransferases and Demethylases Associated with Metastasis, Tumor Invasion, CpG73 Methylation, and HPV Status in Head and Neck Squamous Cell Carcinoma
Source: Curr Issues Mol Biol. 2023 May 27;45(6):4632–46. doi: 10.3390/cimb45060294 (PMC10297334; doi:10.3390/cimb45060294)
Supplement: Supplementary file 1 [file cimb-45-00294-s001.zip › cimb-2320111-supplementary.pdf]

Supplementary information for

Gene Expression Profiles of Methyltransferases and Demethylases Associated with Metastasis, Tumor Invasion, CpG73 Methylation, and HPV Status in Head and Neck Squamous Cell Carcinoma

Larisa Goričan [1,†], Tomaž Büdefeld [1,†], Helena Čelešnik [1,2], Matija Švagan [3], Boštjan Lanišnik [3] and Uroš Potočnik [1,2,4,*]

1 Centre for Human Genetics and Pharmacogenomics, Faculty of Medicine, University of Maribor, 2000 Maribor, Slovenia; larisa.gorican@um.si (L.G.); tomaz.buedefeld@um.si (T.B.)
2 Laboratory for Biochemistry, Molecular Biology and Genomics, Faculty of Chemistry and Chemical Engineering, University of Maribor, 2000 Maribor, Slovenia
3 Department of Otorhinolaryngology, Cervical and Maxillofacial Surgery, University Medical Centre Maribor, Ljubljanska ulica 5, 2000 Maribor, Slovenia
4 Department for Science and Research, University Medical Centre Maribor, 2000 Maribor, Slovenia
* Correspondence: uros.potocnik@um.si; Tel.: +386-2-2345-854
† These authors contributed equally to this work.

Content:

Supplementary Figures S1 – S3

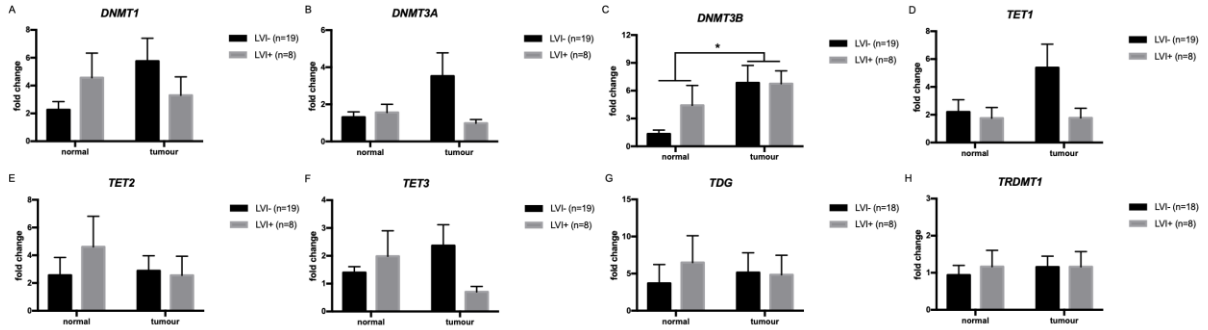

Figure S1: Expression of DNA methyltransferases *DNMT1*, *DNMT3A*, *DNMT3B*, DNA demethylases *TET1*, *TET2*, *TET3* and *TDG* and RNA methyltransferase *TRDMT1* in HNSCC with lymphovascular invasion (LVI+). Differences between groups were examined by two-factor repeated measures ANOVA with tissue type and invasion as independent factors followed by Sidak's multiple comparisons *post hoc* test. The expression of *DNMT3B* was significantly higher in tumour compared to normal tissue irrespective of lymphovascular invasion. For each gene, paired normal tissue corresponding to local tumours (LVI-) was used as a reference. Data are shown as mean +/- S.E.M., $*p < 0.05$

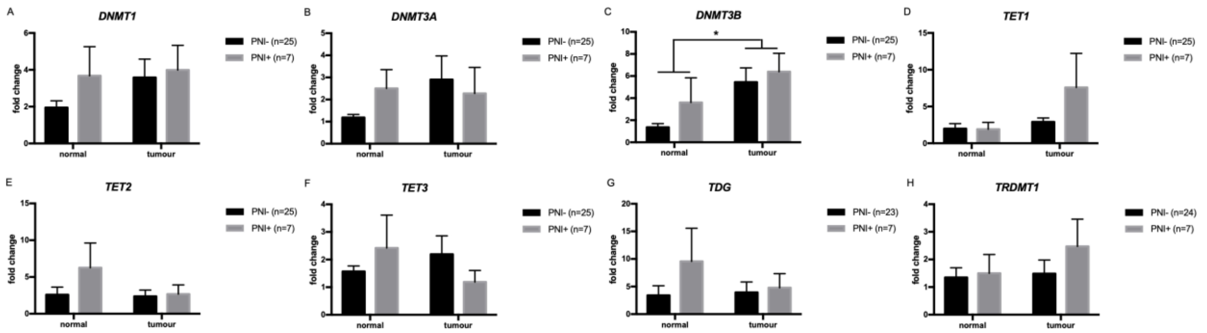

Figure S2: Expression of DNA methyltransferases *DNMT1*, *DNMT3A*, *DNMT3B*, DNA demethylases *TET1*, *TET2*, *TET3* and *TDG* and RNA methyltransferase *TRDMT1* in HNSCC with perineural invasion (PNI+). Differences between groups were examined by two-factor repeated measures ANOVA with tissue type and invasion as independent factors, followed by Sidak's multiple comparisons *post hoc* test. The expression of *DNMT3B* was significantly higher in tumour compared to normal tissue irrespective of perineural invasion. For each gene, paired normal tissue corresponding to local tumours (PNI-) was used as a reference. Data are shown as mean +/- S.E.M., *p < 0.05.

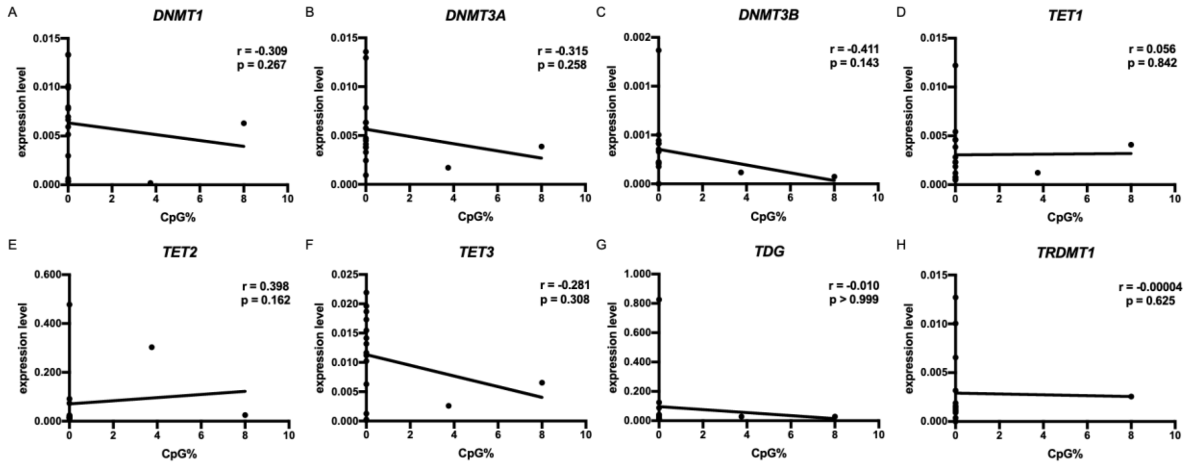

Figure S3: Correlation between CpG73 methylation and mRNA expression of DNA methyltransferases *DNMT1*, *DNMT3A* and *DNMT3B*, DNA demethylases *TET1*, *TET2*, *TET3* and *TDG,* and RNA methyltransferase *TRDMT1* in normal tissue. Outliers were removed using Nalimov test ($\alpha = 0.05$) and the correlation between the gene expression and level of CpG73 methylation was analysed by Spearman's rank-order correlation. r = Spearman's rank correlation coefficient, for all genes: n=18.
